# Direct transport versus inter-hospital transfer of trauma victims in the Brazilian Emergency Medical System

**DOI:** 10.1016/j.tcr.2022.100743

**Published:** 2022-12-05

**Authors:** Fernando Fonseca Costa, Simone de Campos Vieira Abib

**Affiliations:** Federal University of São Paulo, Brazil

**Keywords:** Trauma centers, Trauma, Mortality, Emergency Medical System, Prehospital care

## Abstract

**Introduction:**

The Brazilian medical emergency services presented a significant development due to the investment in emergency care units, increasing the expansion of the services. However, there was a surge in need for secondary patient transfers, which served as the common link in a wide web of tertiary hospital access. This study aimed to assess the outcome of trauma patients who required secondary transfer.

**Patients and methods:**

This prospective observational cross-sectional study included 2302 patients (565 from the study group and 1737 from the control group) and compared the outcome of patients hospitalized for trauma referred by secondary transfer or those who directly visited the Emergency Unit of the municipality with a Brazilian medical emergency system.

**Results:**

As for the trauma mechanism, there was a predominance of blunt trauma (93.32 %), 34.5 % were elderly, 12.45 % suffered severe traumatic brain injury, and 18.44 % had severe trauma rate (injury severity score > 15). The outcome of death did not present a significant difference between the groups, even when evaluated considering possible risk factors, such as the elderly age (over 65 years of age) and trauma index.

**Conclusion:**

There was no significant difference in terms of the outcome of death in patients who underwent secondary transfer and those with direct access to medical emergency services. However, patients who underwent secondary transfer had an increase in the length of hospital stay.

## Highlights

• Impact of direct transport to the trauma center on mortality
• Trauma care in the Brazilian emergency medical system model
• Prehospital decision in the transport of trauma victims
• Consequences related to transfers for the treatment of trauma victims

## Introduction

The recognition of organized systems for the care of trauma victims as an essential policy to improve outcomes is widely disseminated in the literature. Despite this, the world presents a great variability of trauma care models often influenced by issues related to economic conditions [1].

The North American trauma system has clear guidelines for structure and processes, and it is considered a standard to be universally implemented, although it presents some weaknesses [2] and an impractical cost for developing countries. Thus, the search for regional adaptations of the trauma care processes is essential for reducing mortality [3], especially with many deaths caused by trauma disease [4].

Brazil has improved the implementation of the Emergency Medical System in the last two decades, with several public policies promoting a large expansion of pre-hospital care services, although hospital structure did not improve [5]. Thus, since there are no established trauma centers with reference, patient migration in the various levels of complexity of the care system depends on regional management to implement specialized trauma care in general hospitals, making the inter-hospital transfer a fundamental component in the Brazilian Emergency Medical System.

The distribution of trauma patients is a process under investigation, especially regarding triage and transport [6], with inter-hospital transfer having an important impact on the care model [7].

The objective of this study is to evaluate the impact in terms of mortality and length of hospital stay of trauma victims who accessed the trauma referral service by direct transport versus inter-hospital transfer in the Brazilian Emergency Medical System model.

## Patients and methods

### Study design and setting

The Brazilian Medical Emergency System consists of a network of pre-hospital care services, in which the trauma victim rescued by the Mobile Emergency Service is evaluated and referred to the unit best prepared for the complexity of the situation or directly to the hospital of reference for trauma care when necessary.

The patient can also access the services through the emergency care units or those non-hospital emergency medical services with a structure for initial care but without the capacity for surgery. These units can provide initial assistance but have to transfer trauma patients in cases requiring specialized trauma care. Moreover, some small hospitals, especially in smaller cities, can also initiate care but may request the transfer of trauma patients (Table 1).

**Table 1:** Characteristic of the study population according to access to the referral hospital.

	Inter-hospital transfer^a^	Direct access^b^	*p*-Value^c^
Male, n (%)	192 (12.0)	144 (87.9 %)	0.278
Age, mean (SD)	**53 (22.7)**	**47 (21.7)**	**<0.01**
Trauma mechanism, blunt, n (%)	519 (26.2)	1632 (75.3)	0.09
Transport accident, n (%)	**141 (12.7)**	**963 (87.2)**	**0.01**
Fall from standing height, n (%)	**223 (35.7)**	**401 (64.3)**	**0.01**
ISS, category			
ISS <15, n (%)	452 (24.0)	1430 (76.0)	0.821
ISS ≥15, n (%)	113 (27,0)	307 (73.0)	
Glasgow Coma Scale, category			
GCS ≤9, n (%)	35 (20.9)	132 (79.1)	0.101
RTS, mean (SD)	5.9 (1.9)	6 (2)	0.324
Death during hospitalization, n (%)	71 (12.4)	189 (10.8)	0.321
Early death (<24 h)	**9 (1.5)**	**78 (4.3)**	**0.001**
Length of hospitalization, mean (SD)	7.6 (13.1)	6.3 (13)	0.039

Abbreviations: ISS, injury severity score; GCS, Glasgow coma scale; RTS, revised trauma score.

a Patients transferred from other health units.

b Patients transported directly to a trauma referral hospital.

c Mann-Whitney test for continuous variables, chi square test for dichotomous variables, Fisher's test for qualitative variables, and Wilcoxon test after Shapiro–Wilk test, with a significance level of 1 %.

The present study was conducted in the city of São José dos Campos, a municipality with 729,737 inhabitants [8], which has implemented the emergency care model according to the national policy of the Brazilian Ministry of Health [9] in the last decade, structured by six emergency care units, nine ambulances of the Mobile Emergency Service, one small hospital with no surgical center, and one Reference Hospital for emergency care with a structure for specialized trauma care.

A prospective observational evaluation of adult trauma patients who required secondary transport (inter-hospital transport) was performed, comparing them with the control group of trauma patients with direct access to the trauma referral hospital of the municipality during the period from January 1, 2018 to December 31, 2019.

### Data collection and variables

Data collection was performed by the adult trauma team during hospital care, accompanied by a nurse dedicated to patient follow-up within the comprehensive care line for trauma victims [10] of the Hospital Dr. José de Carvalho Florence.

Upon entry into the trauma care room, the following follow-up procedures were performed by surgeons and trauma support specialists: a record of trauma including the trauma mechanism (blunt or penetrating), causes of trauma (transportation accident, interpersonal violence, fall from standing height, and other external causes), types of access to the service (direct or with secondary transport), revised trauma scale (RTS), injury severity score (ISS).

### Outcome

The follow-up visits with a record of the hospitalization outcome allowed the analysis of the outcome of this study (mortality) and the record the length of hospital stay in the number of days.

### Statistical analysis

Group analysis was performed using comparative statistical tests [11], the Chi-square test for qualitative variables and the Mann–Whitney test for numeric dependent variables without normal distribution and for ordinal qualitative data.

The study presents a marginal descriptive analysis of the variables involved and an inferential analysis, using the Fisher test to identify the association between the group variable and the categorical outcome variables, and the Wilcoxon test to study the relationship between the “group” variable and the numeric variable “days of stay”. The Wilcoxon test was used because the numeric variable “days of stay”, according to the Shapiro–Wilk test, at a statistical significance level of 1 %, does not follow a normal distribution (*p* < 0.001).

Finally, a logistic regression model was used for variables related to secondary transport and hospitalization-outcome and linear regression for length of stay.

### Ethical aspects

The research was conducted following the guidelines of the National Health Council on Research Ethics as per Resolution CNS 196/96 Guidelines and Norms Regulating Research Involving Human Beings. This study was registered with the Teaching and Research Committee of the Federal University of the State of São Paulo under No. 0679/2018 (Table 2).

**Table 2:** Results of the logistic regression model for the outcome of death.

Variable^a^	Standard error	t-Value	p-Value	Odds ratio
Male	**0.26**	**2.56**	**0.010**	**1.93**
Age > 59 years	**0.27**	**2.85**	**0.004**	**2.16**
Fall from standing height	**0.29**	**4.12**	**<0,05**	**3.36**
Severe TBI (GCS < 9)	**0.26**	**7.64**	**<0,05**	**7.39**
ISS > 15	**0.25**	**6.23**	**<0.05**	**4.70**
Needed secondary transportation	0.28	0.70	0.487	0.82

Abbreviations: TBI, traumatic brain injury; GCS, Glasgow coma scale; ISS, injury severity score.

a Significant control variables at 10 % in the statistical tests applied to the death outcome were sex, old age (over 65 years of age), transport accident, fall from standing height, severe TBI, and ISS > 15, which were studied in a logistic regression model.

## Results

A total of 2302 patients were included in the study, of which 565 (24.5 %) accessed the referral hospital by inter-hospital transfer, and 1737 (75.4 %) directly visited the emergency department of the hospital.

There was a significant predominance of blunt trauma (93.3 %), and the main cause of trauma was transportation accidents (44,3 %), followed by a fall from standing height (FSH) (27.9 %).

Some severity factors of the victims were considered: 824 (35.7 %) were >59 years old, 256 (11.1 %) suffered a severe traumatic brain injury (TBI) or Glasgow Coma Scale (GSS) <9, and 444 (19.2 %) presented anatomical trauma with an ISS > 15. The patients showed an RTS of 6 and ISS of 9 on average, and the average length of hospital stay was 6 days.

The results of the present study presented 260 deaths (11.2 %), of which 87 (3.7 %) occurred in <24 h (early deaths).

The relationship between the groups and the outcome variable, death, with no control by any other variable, did not present statistical significance according to Fisher's test (*p* = 0.321).

The relationship between the groups and the outcome variable early death, with no control by any other variable, showed statistical significance evaluated by Fisher's test (*p* = 0.001).

The relationship between the groups and the variables length of stay, with no control by any other variable, showed statistical significance (*p* = 0.039), assessed using the Wilcoxon test, since the numeric variable days of stay, according to the Shapiro–Wilk test, at a level of statistical significance of 1 %, does not follow a normal distribution (*p* < 0.001).

The significant control variables at 10 % in the statistical tests applied to the outcome, death, were sex, old age (over 65 years of age), transport accident, FSH, severe TBI, and ISS > 15, which were studied in a logistic regression model. Only the transportation-related accident was not significant in the adjusted model with all the variables chosen and was removed from the model, as the *p*-value referring to the secondary transport variable is >0.1; therefore, with a significance level of 10 %, the chance of a patient who underwent secondary transport dying is equal to that of another patient who did not undergo secondary transport, given that both patients have the same values for the control variables.

Significant control variables at 10 % in the statistical tests applied to the outcome of early death (<24 h) were transport accident, severe TBI, and ISS > 15, which were studied in a logistic regression model.

When assessing the length of stay, only the variables severe TBI and ISS > 15 were significant, and the linear regression model was used, comparing the mean length of stay of an individual who underwent secondary transfer with the mean length of stay of an individual who did not undergo secondary transfer, with the same values in the control variables; this is done using a linear regression model for the logarithm of the days of stay. The comparison is made by the logarithm of the division between the mean times, which is the value estimated by the model. The mean hospital stay of an individual who underwent secondary transfer is 1.37 times the mean hospital stay of another individual who did not undergo secondary transfer, given that both individuals have the same values for the control variables (Table 3).

**Table 3:** Result of the linear regression model for length of hospital stay.

	Estimate	Standard error	t-value	Pr(>|t|)	Exp (est)
Severe TBI (GCS < 9)	0.322	0.096	3.344	0.001	1.380
Severe trauma (ISS > 15)	0.268	0.075	3.602	0.000	1.308
Inter-hospital transfer	**0.129**^a^	**0.065**	**1.984**	**0.048**	**1.137**

a The mean length of stay of an individual who underwent secondary transfer is 1.37 the mean length of stay of another individual who did not undergo secondary transfer, given that both individuals have the same values for the control variables.

## Discussion

This study aims to be a pioneer in research focused on the secondary transport of trauma victims in the Brazilian model of the organization of emergency care, allowing for an analysis of the care system from the perspective of elements known in the literature as influencing the prognosis of these victims.

The current model of care implemented in Brazil works to cover all emergencies, and previous studies focused on trauma system [1]. Thus, including the suggestions of the literature in the local urgency and emergency system should be considered, since, when regularly performed, there was no worsening in mortality in patients requiring secondary transport; however, there was an increase in the length of hospital stay in the analyzed sample, and mortality indicators are below those recommended in countries with developed trauma systems [1] [12], indicating opportunities for improvement.

The cost-effectiveness of centralizing specialized hospital care has been important in units involving >650 critically ill patients (ISS > 15) per year [13]. The reference hospital in this study presented 216 cases (18.44 %) of patients per year in the same condition, which is below the suggested volume, and it is difficult to overcome this metric for the Brazilian reality, especially considering the structural and economic differences involving the development of a health care system network.

Despite the evidence suggesting the importance of the specialized trauma hospital and that the movement to structure this system had begun in 1976 in the United States through the activism of the American College of Surgeons [14], the adequacy of the world scenario to this model was not so fast, even in developed countries, such as the United Kingdom, which occurred only two decades later [15].

A study from the war in Iraq [16] showed that the recording and management of data at all stages of care is of essence in the battlefield and a military section was created especially for this purpose [17]. The present study showed an ineffective transfer communication for the group of patients with secondary transport.

The line of care is a management technique that can be an alternative [18]. In the present study, all patients underwent monitoring and attention of the line of care team for trauma victims, seeking proactive monitoring of care indicators with a focus on comprehensive support to victims of external causes. This management could be adopted in the Brazilian Unified Health System; thus, the implementation of a model based on this technology would enable the use of installed structure with the inclusion of processes focused on specialized care to trauma victims, including tracking and monitoring of specific indicators, as well as the institution of a database that follows the patient at all levels of care.

A large observational study showed data of patients transported by the U.S. Air Force from Balad airbase in Iraq to Landstyhl Hospital in Germany, and its results corroborate that the safety in the mobility of critical victims for long distances, including the rapid movement of patients from the battlefront to the place of definitive treatment contributes to reducing mortality [17].

In the analysis of the difference in the distribution of variables in the independent groups, individuals who underwent secondary transportation (study group) and individuals who had direct access to a tertiary hospital (control group) showed a significant difference in age (*p* < 0.01), although there was no difference between the type of trauma as open or closed (*p* = 0.09), there was a difference as to the causes of trauma, and in the study group there were more victims of FSH in relation to the control group, while in the control group there were more victims of transport accidents. Despite these differences between the study groups, the severity of the victims in both groups showed no statistically significant difference using the RTS severity scale (*p* = 0.324), ISS (*p* = 0.82), and GCS (*p* = 0.101), corroborating the comparative feasibility between the groups.

Although secondary transport did not show an increase in mortality from trauma, the limitations of the study do not allow us to affirm the safety of this model.

Thus, the implementation of protocols for direct access to the specialized trauma center [6] associated with unified trauma registries are challenges for the Brazilian Emergency Medical System aiming at international best practices [1].

The main limitation of this study was not evaluating the trauma victims who had primary access outside the reference hospital and had unfavorable progress associated with non-directly evaluation on the care provided before admission to the trauma reference hospital.

## Conclusions

To date, the present study is the first to investigate the care of trauma victims from the perspective of inter-hospital transport in the Emergency Medical System of Brazil, where this is an essential procedure due to the vast territory and the disproportionality between pre-hospital and hospital care.

In the sample study, inter-hospital transport did not cause worsening of the outcome, death; therefore, it did not impact mortality but was associated with an increased length of hospital stay.

The emergency system adopted in Brazil is based on complex references and presents the need for secondary transport. However, it does not include a specific system for trauma. The results of this study suggest the feasibility of patient transfers between the system components, but due to the worsening of a secondary outcome (worsening of mortality in <24 h and with increased length of hospital stay), further studies are warranted to determine the necessary adaptation of parameters of internationally recognized systems in the literature to the reality of the Brazilian model.
